# The association between different hypoglycemic regimens and postoperative diabetic macular edema after vitrectomy in the Japanese patients with proliferative diabetic retinopathy

**DOI:** 10.3389/fendo.2022.764254

**Published:** 2022-07-22

**Authors:** Chunyan Lei, Yun Zhang, Meixia Zhang

**Affiliations:** ^1^ Department of Ophthalmology, West China Hospital, Sichuan University, Chengdu, China; ^2^ Research Laboratory of Macular Disease, West China Hospital, Sichuan University, Chengdu, China

**Keywords:** diabetic macular edema, proliferative diabetic retinopathy, pars plana vitrectomy, hypoglycemic regimens, insulin

## Abstract

**Purpose:**

To study the association between different hypoglycemic regimens and postoperative diabetic macular edema (DME).

**Methods:**

A secondary analysis based on a retrospective cohort study.

**Results:**

In this secondary analysis, 124 eyes from patients with proliferative diabetic retinopathy (PDR) who underwent pars plana vitrectomy (PPV) between January 2008 and September 2012 were included. We found that compared with oral hypoglycemic medication, oral hypoglycemic medication plus insulin treatment revealed an insignificant relationship with postoperative DME (odds ratio [OR]=0.8, 95% confidence interval [CI]: 0.12-5.21, P=0.8167), only insulin treatment revealed a significant association with postoperative DME (OR=0.10, 95% CI: 0.01-0.84, P=0.0337) after adjusted age, sex. After adjusted age, sex, diabetes mellitus (DM) duration, glycosylated hemoglobin (HbA1c), the results did not have obvious changes (OR=0.61, 95% CI: 0.09-4.26, P=0.6187; OR=0.07, 95% CI: 0.01-0.65, P=0.0197). Furthermore, after adjusted age, sex, DM duration, HbA1c, hypertension, intraoperative retinal photocoagulation, vitreous hemorrhage, macular detachment, fibrovascular membrane, intraocular lens implantation and microincision vitrectomy surgery, the results were consistent (OR=0.66, 95% CI: 0.05-9.49, P=0.7621; OR=0.06, 95% CI: 0.00-0.81, P=0.0342). The same trend was observed in these adjusted models as well (p for trend was 0.0254, 0.0141, and 0.0311, respectively).

**Conclusion:**

In conclusion, our results of the secondary analysis should be interpreted as a significant association between insulin treatment and reduced risks of postoperative DME in Japanese PDR patients with PPV surgery, compared with oral medications. Well glycemic control with longstanding insulin therapy may be beneficial to reduce the risks of postoperative DME in PDR patients. Our investigation calls for large-scale and long-term prospective clinical studies for a full evaluation of the exact role of insulin in the progression of postoperative DME.

## Introduction

Diabetic retinopathy (DR) is the primary cause of visual impairment and blindness among working-age individuals in developed countries (1). Two important factors affecting the vision of DR patients are complications associated with proliferative diabetic retinopathy (PDR) and diabetic macular edema (DME), which are thought to occur as a result of vascular endothelial growth factor (VEGF) and other cytokines into the vitreous cavity (2, 3). PDR, the most advanced stage of DR, is characterized by neovascularization and proliferative membrane formation, which may cause vitreous hemorrhage and tractional retinal detachment (4–7). Those patients with PDR often require pars plana vitrectomy (PPV) treatment and emergency management to prevent further vision loss. Although DME can occur at any DR stage, the prevalence of DME was associated with diabetes mellitus (DM) duration and DR severity. PDR and DME frequently occur together and the prevalence of DME in PDR patients varied from 30% to 72.6% (8, 9). The risk factors for DME have been widely studied, including duration of diabetes (10), hypertension (11, 12), glycosylated hemoglobin (HbA1c) level (13–15), insulin (15–19), and other factors (11).

There were divergent findings of the association between different hypoglycemic regimens and DME. Several independent clinical studies (14–16, 18–21) and meta-analysis (17) confirmed that insulin use increased the risks of DME in patients with type 2 diabetes mellitus (T2DM) compared with oral hypoglycemic agents. What’s more, this finding was supported by another experimental research that insulin increased retinal vascular permeability in diabetic mice (22). While several studies pointed to a potential effect of insulin on reducing risks of DME (23, 24). The stable glycemic control induced by insulin might be one possible explanation. Insulin itself has a weak to moderate stimulatory effect on the proliferation of human retinal pigment epithelium (RPE) cells and could promote RPE wound healing (25), which might be another possible explanation. Apart from insulin, different oral hypoglycemic agents may have different effects on DME. Thiazolidinediones (TZDs) including pioglitazone and rosiglitazone, are insulin-sensitizing medications that can be used for glycemic control in T2DM. A growing body of studies has highlighted that TZDs might contribute to increase risks of DME (26–29). In addition to the aforementioned, the association between sulphonylureas, metformin, and the risk of DME has also been investigated (14, 30).

Treatments for DME have gradually evolved from the initial grid macular coagulations to intravitreal injection including triamcinolone, Ozurdex, and anti-VEGF agents. In addition to the aforementioned, PPV surgery is proven to be associated with structural benefits compared with the natural history of DME (9, 31, 32). PPV treatment in PDR aims at removing vitreoretinal traction and vitreous hemorrhage, clearing various cytokines from the vitreous cavity, reattaching detached neuroretina, maintaining media transparency, and improving ocular circulation (7, 33). Even if patients with PDR have undergone PPV treatment, some patients still develop postoperative DME. How to make those patients with postoperative DME preserve their vision and improve their quality of life is a problem that needs to be solved urgently. In addition to investigating the specific treatments of postoperative DME, more attention we should pay to the risk factors of postoperative DME. However, first-hand clinical data regarding the role of different hypoglycemic regimens in postoperative DME is scarce. In this study, we performed a secondary data analysis based on existing data that comes from the published paper (34) to investigate the association between different hypoglycemic regimens and postoperative DME.

## Materials and methods

### Data source

We freely downloaded the raw data uploaded by Nishi et al. (34) from the **Supplementary Materials** in PLOS One. Since Nishi et al. (34) have authorized the ownership of the original data to PLOS One, we can use this data to perform secondary data analysis based on different scientific assumptions. Data from: Factors correlated with visual outcomes at two and four years after vitreous surgery for proliferative diabetic retinopathy (PMID: 33444332).

### Study population

Nishi et al. (34) completed the entire study. The specific details were described in the original paper reported by Nishi et al. (34). They conducted a retrospective cohort study at Yamagata University Hospital, Yamagata, Japan between January 2008 and September 2012. They retrospectively reviewed the medical records of these patients.

A total of 128 eyes were collected from the PDR patients who had been to Yamagata University Hospital or other hospitals after three-port 20-gauge (G) PPV or microincision vitreous surgery (MIVS) (23-G or 25-G). All patients with persistent vitreous hemorrhage and tractional retinal detachment and two vitreoretinal surgeons performed all the surgical procedures. However, surgical cases of only DME were excluded. All patients did not receive anti-VEGF therapy as a preoperative adjunct. Pan retinal photocoagulation was cautiously performed before or during PPV on all patients. Participants with vision-affecting lesions such as posterior capsular opacification, progressed cataract, neovascular glaucoma, and DME during the postoperative course were treated. The follow-up time was 3 months, 6 months, 1 year, 2 years, and 4 years after the primary PPV.

This study was performed by Japanese researcher Nishi et al. at the Yamagata University Hospital, Yamagata, Japan. In the previously published article (34), Nishi et al. have clearly stated that the study was performed following the Declaration of Helsinki and approved by the Ethics Committee of the Yamagata University Faculty of Medicine (approval number: H26-21). All data were fully anonymized before we accessed them and the institutional review board waived the requirement for informed consent.

### Variables

The systemic factors collected were as follows: age, sex, duration from visual loss awareness to the primary vitreous surgery, hypertension history, DM duration, preoperative HbA1c, oral hypoglycemic medication, insulin treatment, diabetic nephropathy history, coronary heart disease and/or stroke history, anticoagulant and/or antiplatelet agent administration, preoperative systolic and diastolic blood pressure, and blood biochemical examination, including blood urea nitrogen, creatinine, estimated glomerular filtration rate (eGFR), uric acid, triglyceride, total cholesterol, hemoglobin.

Moreover, the ophthalmologic findings were categorized into three sections: preoperative, intraoperative, and postoperative. The preoperative ophthalmologic findings were as follows: intraocular lens implantation, retinal photocoagulation, the history of intravitreal injection of triamcinolone acetonide, rubeosis iridis, ocular hypertension (>21 mmHg), vitreous hemorrhage, posterior vitreous detachment, fibrovascular membrane, retinal detachment, and macular detachment. The intraoperative ophthalmologic findings were the following: cataract surgery, intraoperative retinal photocoagulation, gas tamponade, silicone oil tamponade, intraoperative complications (iatrogenic retinal break and retinal dialysis), and the number of used gauges (20-G or MIVS). Lastly, the postoperative ophthalmologic findings were as follows: reoperation and postoperative complications (vitreous hemorrhage, retinal detachment, DME, and neovascular glaucoma).

### Statistical analysis

Demographic characteristics and study outcomes were summarized using descriptive statistics. Continuous variables were summarized with mean ± standard deviations (SD) and categorical variables with percentages. Two dichotomous categorical variables (“oral DM medication” and “insulin treatment”) were classified into a quartile categorical variable: no medication, only oral medication, oral medication plus insulin, only insulin. To improve the statistical power, of the 128 eyes, 4 eyes without any medication were excluded from this study. We first compared the data distribution of each covariate among the three different hypoglycemic regimens using the t-test (normal distribution) or Kruskal-Wallis rank-sum test (non-normal distribution) for continuous variables and χ^2^ tests for categorical data and *post-hoc* comparison was performed (**Table 1**). Next, univariate logistic regression (**Table 2**) and multivariate logistic regression models (**Table 3**) were used to examine the association between different hypoglycemic regimens and postoperative DME. Test for trend was used to observe the trend change between different hypoglycemic regimens and postoperative DME in different adjusted models (**Table 3**). Subgroup analysis (**Table 4**) was used to see whether the results were stable at all stratifications. Statistical analyses were performed using Empower Stats (http://www.empowerstats.com; X&Y Solutions Inc., Boston, MA) and R software, version 3.4.3 (http://www.R-project.org/, The R Foundation). A two-sided P < 0.05 was considered to be statistically significant.

**Table 1:** Baseline characteristics of participants.

	Total	Oral medication Only (1)	Oral medication + insulin (2)	Insulin only (3)	P	*Post-hoc*		
						2 vs. 1 (MD; P)	3 vs. 1 (MD; P)	3 vs. 2 (MD; P)
**No. (eyes)**	124	48	16	60				
Age (years)	55.90 ± 11.36	55.35 ± 11.60	56.25 ± 8.41	56.23 ± 11.97	0.916	0.8958; 0.9603	0.8792; 0.9169	-0.0167; 0.9999
Male sex	87 (70.16%)	40 (83.33%)	5 (31.25%)	42 (70.00%)	<0.001	-0.5208; 0.0002	-0.1333; 0.2541	0.3875; 0.0053
Duration from visual loss awareness to the primary surgery (months)	4.00 ± 6.32	3.15 ± 4.12	3.62 ± 4.54	4.78 ± 7.96	0.399	0.4792; 0.9628	1.6375; 0.3776	1.1583; 0.792
Diabetes mellitus duration (years)	12.18 ± 8.46	10.54 ± 7.30	12.81 ± 7.57	13.32 ± 9.41	0.227	2.2708; 0.6199	2.7750; 0.2088	0.5042; 0.9754
SBP (mmHg)	138.65 ± 22.59	138.69 ± 24.84	139.00 ± 17.13	138.53 ± 22.31	0.997	0.3125; 0.9988	-0.1542; 0.9993	-0.4667; 0.9971
DBP (mmHg)	78.66 ± 14.30	79.48 ± 13.25	80.31 ± 14.64	77.57 ± 15.15	0.7	0.8333; 0.9780	-1.9125; 0.7716	-2.7458; 0.7763
**Systemic diseases**								
Hypertension	76 (61.29%)	33 (68.75%)	13 (81.25%)	30 (50.00%)	0.03	0.125; 0.6387	-0.1875; 0.1113	-0.3125; 0.0569
Diabetic nephropathy	84 (67.74%)	35 (72.92%)	12 (75.00%)	37 (61.67%)	0.37	0.0208; 0.9871	-0.1125; 0.4335	-0.1333; 0.5723
History of coronary heart disease and/or stroke	22 (17.74%)	8 (16.67%)	0 (0.00%)	14 (23.33%)	0.092	-0.1667; 0.2840	0.0667; 0.6364	0.2333; 0.0775
**Systemic medication history**								
Anticoagulant and/or antiplatelet agent administration	28 (22.58%)	10 (20.83%)	1 (6.25%)	17 (28.33%)	0.16	-0.1458; 0.4487	0.075; 0.6232	0.2208; 0.1482
**Preoperative ophthalmologic findings**								
Intraocular lens implantation	38 (30.65%)	14 (29.17%)	4 (25.00%)	20 (33.33%)	0.781	-0.0417; 0.9485	0.0417; 0.8891	0.0833; 0.8007
Preoperative retinal photocoagulation	109 (87.90%)	44 (91.67%)	13 (81.25%)	52 (86.67%)	0.499	-0.1042; 0.5164	-0.05; 0.7120	0.0542; 0.8276
Intravitreal injection of TA	1 (0.81%)	0 (0.00%)	0 (0.00%)	1 (1.67%)	0.584	-0.0000; 1.0000	0.0167; 0.6068	0.0167; 0.7887
Rubeosis iridis	16 (12.90%)	3 (6.25%)	1 (6.25%)	12 (20.00%)	0.074	-0.0000; 1.0000	0.1375; 0.08657	0.1375; 0.3083
Ocular hypertension	10 (8.06%)	5 (10.42%)	1 (6.25%)	4 (6.67%)	0.746	-0.0417; 0.8593	-0.0375; 0.7615	0.0042; 0.9984
Vitreous hemorrhage	98 (79.03%)	41 (85.42%)	15 (93.75%)	42 (70.00%)	0.044	0.0833; 0.7529	-0.1542; 0.1210	-0.2375; 0.0937
Posterior vitreous detachment	31 (25.00%)	16 (33.33%)	3 (18.75%)	12 (20.00%)	0.233	-0.1458; 0.4756	-0.1333; 0.2541	0.0125; 0.9942
Fibrovascular membrane	71 (57.26%)	27 (56.25%)	8 (50.00%)	36 (60.00%)	0.76	-0.0625; 0.9018	0.0375; 0.9206	0.1000; 0.7573
Retinal detachment	30 (24.19%)	12 (25.00%)	4 (25.00%)	14 (23.33%)	0.977	-0.0000; 1.0000	-0.0167; 0.9785	-0.0167; 0.9898
Macular detachment	18 (14.52%)	7 (14.58%)	1 (6.25%)	10 (16.67%)	0.576	-0.0833; 0.6956	0.0208; 0.9507	0.1042; 0.5514
**Intraoperative ophthalmologic findings**								
Cataract surgery	60 (48.39%)	19 (39.58%)	11 (68.75%)	30 (50.00%)	0.122	0.2917; 0.1090	0.1042; 0.5272	-0.1875; 0.3758
Intraoperative retinal photocoagulation	107 (86.29%)	43 (89.58%)	14 (87.50%)	50 (83.33%)	0.637	-0.0208; 0.9764	-0.0625; 0.6222	-0.0417; 0.9045
Gas tamponade	24 (19.35%)	8 (16.67%)	1 (6.25%)	15 (25.00%)	0.201	-0.1042; 0.6324	0.0833; 0.5220	0.1875; 0.2139
Silicone oil tamponade	3 (2.42%)	3 (6.25%)	0 (0.00%)	0 (0.00%)	0.088	-0.0625; 0.3338	-0.0625; 0.0906	0.0000; 1.0000
Intraoperative complications	15 (12.10%)	5 (10.42%)	2 (12.50%)	8 (13.33%)	0.898	0.0208; 0.9740	0.0292; 0.8915	0.0083; 0.9956
MIVS	55 (44.35%)	26 (54.17%)	8 (50.00%)	21 (35.00%)	0.122	-0.0417; 0.9541	-0.1917; 0.1160	-0.1500; 0.5292
**Postoperative ophthalmologic findings**								
Reoperation	22 (17.74%)	10 (20.83%)	2 (12.50%)	10 (16.67%)	0.718	-0.0833; 0.7351	-0.0417; 0.8426	0.0417; 0.9220
Postoperative NVG	6 (4.84%)	2 (4.17%)	1 (6.25%)	3 (5.00%)	0.942	0.0208; 0.9409	0.0083; 0.9786	-0.0125; 0.9772
Postoperative DME	10 (8.06%)	7 (14.58%)	2 (12.50%)	1 (1.67%)	0.039	-0.0208; 0.9609	-0.1292; 0.0378	-0.1083; 0.3263
**Laboratory data**								
HbA1c (%)	7.47 ± 1.55	7.25 ± 1.35	7.61 ± 1.25	7.61 ± 1.76	0.454	0.3604; 0.7026	0.3625; 0.4541	0.0021; 1.00
BUN (mg/dl)	21.24 ± 11.63	22.31 ± 10.71	19.88 ± 5.52	20.75 ± 13.46	0.696	-2.4375; 0.7508	-1.5625; 0.7696	0.8750; 0.9618
Crea (mg/dl)	1.38 ± 1.47	1.69 ± 1.82	0.83 ± 0.27	1.27 ± 1.29	0.093	-0.8592; 0.1054	-0.4274; 0.2860	0.4318; 0.5434
eGFR (ml/min/1.73 m^2^)	63.75 ± 37.11	56.77 ± 30.53	68.49 ± 23.32	68.06 ± 43.88	0.252	11.7271; 0.5170	11.2983; 0.2593	-0.4288; 0.9991
UA (mg/dl)	5.70 ± 1.53	5.92 ± 1.34	5.52 ± 1.58	5.57 ± 1.65	0.433	-0.4062; 0.6290	-0.3533; 0.46	0.0529; 0.9917
TG (mg/dl)	175.33 ± 128.92	192.54 ± 144.09	158.88 ± 74.33	165.95 ± 127.67	0.492	-33.6667; 0.6398	-26.5917; 0.5390	7.0750; 0.9793
TC (mg/dl)	205.10 ± 48.33	207.79 ± 50.12	225.50 ± 55.63	197.50 ± 43.64	0.106	17.07083; 0.4078	-10.2917; 0.5091	-28.0000; 0.0982
Hb (g/dl)	12.76 ± 2.02	12.56 ± 1.61	12.24 ± 2.51	13.06 ± 2.16	0.243	-0.3208; 0.8458	0.4983; 0.4105	0.8192; 0.3211

2 vs. 1: Oral medication + insulin group vs. Oral medication only group; 3 vs. 1: Insulin only group vs. Oral medication only group; 3 vs. 2: Insulin only group vs. Oral medication + insulin group; MD, mean difference; MIVS, microincision vitrectomy surgery; NVG, neovascular glaucoma; DME, diabetic macular edema; HbA1c, glycosylated hemoglobin; SBP, systolic blood pressure; DBP, diastolic blood pressure; BUN, blood urea nitrogen; Crea, creatinine; eGFR, estimated glomerular filtration rate; UA, uric acid; TA, triamcinolone acetonide; TG, triglyceride; TC, total cholesterol; Hb, hemoglobin.

**Table 2:** The results of univariate analysis.

	Postoperative DME	P
	OR (95% CI)	
Age, per 1 year increase	0.97 (0.92, 1.03)	0.3114
Male vs female	0.99 (0.24, 4.07)	0.9907
Duration from visual loss awareness to the primary surgery, per 1-month increase	1.00 (0.90, 1.11)	0.9582
Diabetes mellitus duration, per 1 year increase	1.01 (0.94, 1.09)	0.8379
SBP, pre 1 mmHg increase	1.01 (0.98, 1.04)	0.4874
DBP, per 1 mmHg increase	1.02 (0.98, 1.07)	0.3078
**Systemic diseases**		
Hypertension vs absent	0.61 (0.17, 2.21)	0.4483
Diabetic nephropathy vs absent	2.00 (0.40, 9.88)	0.3951
History of coronary heart disease and/or stroke vs absent	0.00 (0.00, Inf)	0.9906
**Systemic medication history**		
Anticoagulant and/or antiplatelet agent administration vs absent	0.85 (0.17, 4.23)	0.8389
**Treatment**		
Oral medication only	Ref	
Oral medication + insulin	0.84 (0.16, 4.51)	0.8357
Insulin only	0.10 (0.01, 0.84)	0.0338
**Preoperative ophthalmologic findings**		
Intraocular lens implantation vs absent	0.00 (0.00, Inf)	0.992
Preoperative retinal photocoagulation vs absent	inf. (0.00, Inf)	0.9923
Intravitreal injection of triamcinolone acetonide vs absent	inf. (0.00, Inf)	0.9901
Rubeosis iridis vs absent	0.73 (0.09, 6.21)	0.776
Ocular hypertension vs absent	0.00 (0.00, Inf)	0.9937
Vitreous hemorrhage vs absent	inf. (0.00, Inf)	0.9934
Posterior vitreous detachment vs absent	1.32 (0.32, 5.44)	0.7041
Fibrovascular membrane vs absent	0.16 (0.03, 0.80)	0.0258
Retinal detachment vs absent	0.33 (0.04, 2.68)	0.297
Macular detachment vs absent	0.00 (0.00, Inf)	0.9915
**Intraoperative ophthalmologic findings**		
Cataract surgery vs absent	1.67 (0.45, 6.22)	0.4473
Intraoperative retinal photocoagulation vs absent	1.47 (0.17, 12.40)	0.7236
Gas tamponade vs absent	0.44 (0.05, 3.65)	0.4464
Silicone oil tamponade vs absent	0.00 (0.00, Inf)	0.9918
Intraoperative complications vs absent	3.64 (0.83, 15.99)	0.0867
MIVS vs absent	1.28 (0.35, 4.67)	0.7084
**Postoperative ophthalmologic findings**		
Reoperation vs absent	1.17 (0.23, 5.95)	0.8456
Postoperative NVG vs absent	0.00 (0.00, Inf)	0.9925
**Laboratory data**		
HbA1c, per 1% increase	1.17 (0.80, 1.71)	0.4187
BUN, per 1 mg/dl increase	1.00 (0.99, 1.02)	0.8614
Crea, per 1 mg/dl increase	0.97 (0.91, 1.05)	0.4578
eGFR, per 1 ml/min/1.73 m2 increase	0.96 (0.60, 1.55)	0.8804
UA, per 1 mg/dl increase	1.00 (0.98, 1.01)	0.6505
TG, per 1 mg/dl increase	1.02 (0.67, 1.56)	0.9337
TC, per 1 mg/dl increase	1.00 (1.00, 1.01)	0.0711
Hb, per 1 g/dl increase	1.13 (0.81, 1.58)	0.4699

MIVS, microincision vitrectomy surgery; NVG, neovascular glaucoma; DME, diabetic macular edema; HbA1c, glycosylated hemoglobin; SBP, systolic blood pressure; DBP, diastolic blood pressure; BUN, blood urea nitrogen; Crea, creatinine; eGFR, estimated glomerular filtration rate; UA, uric acid; TG, triglyceride; TC, total cholesterol; Hb, hemoglobin.

OR, odds ratio; CI, confidence interval; Ref, reference.

**Table 3:** Relationship between different hypoglycemic regimens and postoperative diabetic macular edema in different models.

Treatment	Adjusted I model (OR, 95% CI, P)	Adjusted II model (OR, 95% CI, P)	Adjusted III model (OR, 95% CI, P)
Oral medication only	Ref	Ref	Ref
Oral medication + insulin	0.80 (0.12, 5.21) 0.8167	0.61 (0.09, 4.26) 0.6187	0.66 (0.05, 9.49) 0.7621
Insulin	0.10 (0.01, 0.84) 0.0337	0.07 (0.01, 0.65) 0.0197	0.06 (0.00, 0.81) 0.0342
P for trend	0.0254	0.0141	0.0311

Adjusted model I adjust for: age; sex.

Adjusted model II adjust for: age; sex; diabetes mellitus duration; glycosylated hemoglobin.

Adjusted model III adjust for: age; sex; diabetes mellitus duration; HbA1c; hypertension; intraoperative retinal photocoagulation; vitreous hemorrhage; macular detachment; fibrovascular membrane; intraocular lens implantation, microincision vitrectomy surgery.

OR, odds ratio; CI, confidence interval, Ref, reference.

**Table 4:** The results of stratified analysis between different hypoglycemic regimens and postoperative diabetic macular edema.

	N	Oral medication only	Oral medication + Insulin	Insulin Only
			OR (95% CI) P	OR (95% CI) P
Age (years)				
<60	74	Ref	5.48 (0.09, 331.15) 0.4162	0.03 (0.00, 1.53) 0.0793
>=60	50	Ref	0.00 (0.00, Inf) 0.9991	Inf. (0.00, Inf) 0.9999
Sex				
Female	37	Ref	0.00 (0.00, Inf) 0.9999	0.00 (0.00, Inf) 0.9997
Male	87	Ref	31.30 (0.33, 2996.74) 0.1390	0.02 (0.00, 1.41) 0.0716
Diabetes mellitus duration (years)				
<10	41	Ref	0.01 (0.00, Inf) 1.0000	0.00 (0.00, Inf) 0.9999
>=10	83	Ref	0.73 (0.04, 11.85) 0.8242	0.05 (0.00, 0.91) 0.0426
HbA1c (%)				
<8	87	Ref	4.90 (0.22, 106.91) 0.3120	0.04 (0.00, 1.24) 0.0668
>=8	37	Ref	0.00 (0.00, Inf) 0.9999	0.00 (0.00, Inf) 0.9997
**Systemic diseases**				
Hypertension				
No	48	Ref	0.00 (0.00, Inf) 0.9998	0.00 (0.00, Inf) 0.9995
Yes	76	Ref	4.23 (0.00, 52444.47) 0.7645	0.00 (0.00, Inf) 0.9969
Diabetic nephropathy				
No	40	Ref	inf. (0.00, Inf) 0.9997	0.00 (0.00, Inf) 0.9999
Yes	84	Ref	0.50 (0.01, 21.49) 0.7191	0.05 (0.00, 1.03) 0.0525
History of coronary heart disease and/or stroke				
No	102	Ref	0.36 (0.03, 4.86) 0.4451	0.06 (0.00, 1.01) 0.0511
Yes	22	Ref	NA	1.00 (0.00, Inf) 1.0000
**Systemic medication history**				
Anticoagulant and/or antiplatelet agent administration				
No	96	Ref	0.42 (0.03, 6.06) 0.5206	0.08 (0.00, 1.56) 0.0952
Yes	28	Ref	inf. (0.00, Inf) 0.9999	inf. (0.00, Inf) 0.9999
**Preoperative ophthalmologic findings**				
Intraocular lens implantation				
No	86	Ref	0.50 (0.04, 6.09) 0.5865	0.06 (0.00, 0.79) 0.0323
Yes	38	Ref	1.00 (0.00, Inf) 1.0000	1.00 (0.00, Inf) 1.0000
Preoperative retinal photocoagulation				
No	15			
Yes	109	Ref	0.72 (0.05, 9.85) 0.8075	0.09 (0.01, 1.27) 0.0738
Intravitreal injection of triamcinolone acetonide				
No	123	Ref	0.16 (0.01, 2.92) 0.2157	0.00 (0.00, Inf) 0.9960
Yes	1			
Rubeosis iridis				
No	108	Ref	0.16 (0.01, 2.92) 0.2157	0.00 (0.00, Inf) 0.9965
Yes	16	Ref	1.00 (0.00, Inf) 1.0000	1.00 (0.00, Inf) 1.0000
Ocular hypertension				
No	114	Ref	0.60 (0.05, 7.40) 0.6886	0.06 (0.00, 0.92) 0.0435
Yes	10			
Vitreous hemorrhage				
No	26			
Yes	98	Ref	0.50 (0.04, 6.09) 0.5865	0.06 (0.00, 0.79) 0.0323
Posterior vitreous detachment				
No	93	Ref	0.95 (0.05, 16.75) 0.9740	0.10 (0.00, 2.49) 0.1594
Yes	31	Ref	0.00 (0.00, Inf) 0.9994	0.00 (0.00, Inf) 0.9994
Fibrovascular membrane				
No	53			
Yes	71	Ref	inf. (0.00, Inf) 0.9998	225.85 (0.00, Inf) 1.0000
Retinal detachment				
No	94	Ref	0.29 (0.01, 5.53) 0.4083	0.06 (0.00, 0.85) 0.0375
Yes	30	Ref	1.00 (0.00, Inf) 1.0000	1.00 (0.00, Inf) 1.0000
Macular detachment				
No	106	Ref	0.50 (0.04, 6.09) 0.5865	0.06 (0.00, 0.79) 0.0323
Yes	18			
**Intraoperative ophthalmologic findings**				
Cataract surgery				
No	64	Ref	0.00 (0.00, Inf) 1.0000	0.00 (0.00, Inf) 0.9997
Yes	60	Ref	1.40 (0.06, 30.67) 0.8313	0.13 (0.00, 4.04) 0.2419
Intraoperative retinal photocoagulation				
No	17	Ref	0.00 (0.00, Inf) 1.0000	0.00 (0.00, Inf) 1.0000
Yes	107	Ref	0.16 (0.01, 2.92) 0.2157	0.00 (0.00, Inf) 0.9963
Gas tamponade				
No	100	Ref	1.37 (0.09, 21.42) 0.8226	0.09 (0.01, 1.38) 0.0835
Yes	24	Ref	0.01 (0.00, Inf) 1.0000	0.00 (0.00, Inf) 0.9999
Silicone oil tamponade				
No	121	Ref	0.50 (0.04, 6.09) 0.5865	0.06 (0.00, 0.79) 0.0323
Yes	3			
MIVS				
No	69	Ref	0.00 (0.00, 0.00) <0.0001	0.00 (0.00, 0.00) <0.0001
Yes	55	Ref	0.33 (0.00, 21.56) 0.6004	0.00 (0.00, Inf) 0.9975
**Postoperative ophthalmologic findings**				
Reoperation				
No	102	Ref	1.37 (0.08, 24.65) 0.8316	0.07 (0.00, 1.09) 0.0574
Yes	22	Ref	0.00 (0.00, Inf) 0.9999	0.01 (0.00, Inf) 1.0000
Postoperative NVG				
No	118	Ref	0.71 (0.05, 9.32) 0.7963	0.06 (0.00, 0.78) 0.0319
Yes	6			
Postoperative complications				
No	94	Ref	42.11(0.66, 2685.01) 0.0777	0.06 (0.00, 3.25) 0.1646
Yes	30	Ref	0.00 (0.00, Inf) 0.9997	0.00 (0.00, Inf) 1.0000

MIVS, microincision vitrectomy surgery; NVG, neovascular glaucoma; HbA1c, glycosylated hemoglobin; OR, odds ratio; CI, confidence interval; Ref, reference.

## Results

### Baseline characteristics of participants

A total of 124 eyes with PDR were enrolled in the final analysis. These participants were divided into three groups according to different hypoglycemic regimens. The average age of the participants was 55.90  ±  11.36 years old, and about 70.16% of them were men. There was no statistically significant difference in age among different hypoglycemic regimens. Patients taking insulin with or without other medications at baseline had longer DM duration (12.81 ± 7.57 and 13.32 ± 9.41 years, respectively) compared with those only taking oral medications (10.54 ± 7.30 years), yet there was no statistical difference. The same trend was observed in the HbA1c level. Other baseline characteristics are listed in **Table 1**.

### Univariate analysis

The results of univariate analysis revealed a significant association between only insulin group and postoperative DME (odds ratio [OR]=0.10, 95% confidence interval [CI]: 0.01-0.84, P=0.0338), compared with only oral medication. There was a statistically significant difference between fibrovascular membrane and postoperative DME (OR=0.16, 95% CI: 0.03-0.80, P=0.0258). We found that other factors were not associated with postoperative DME. The results of univariate analysis are shown in **Table 2**.

### The relationship between different hypoglycemic regimens and postoperative DME

We used a logistic regression model to evaluate the associations between different hypoglycemic regimens and postoperative DME (**Table 3**). Meanwhile, we showed three adjusted models in **Table 3**. In adjusted model I (adjusted age, sex), compared with oral hypoglycemic medication, oral hypoglycemic medication plus insulin treatment revealed no association with postoperative DME (OR=0.8, 95% CI: 0.12-5.21, P=0.8167), while only insulin treatment revealed a significant association with postoperative DME (OR=0.10, 95% CI: 0.01-0.84, P=0.0337). In adjusted model II (adjusted age, sex, DM duration, HbA1c), the results did not have obvious changes (OR=0.61, 95% CI: 0.09-4.26, P=0.6187; OR=0.07, 95% CI: 0.01-0.65, P=0.0197). Furthermore, in adjusted model III (adjusted age, sex, DM duration, HbA1c, hypertension, intraoperative retinal photocoagulation, vitreous hemorrhage, macular detachment, fibrovascular membrane, intraocular lens implantation, MIVS), the results were consistent (OR=0.66, 95% CI: 0.05-9.49, P=0.7621; OR=0.06, 95% CI: 0.00-0.81, P=0.0342). The same trend was observed in these adjusted models as well (p for trend was 0.0254, 0.0141, and 0.0311, respectively).

### The results of stratified analysis between different hypoglycemic regimens and postoperative DME

Each stratification was adjusted for all the factors (age, sex, DM duration, HbA1c, hypertension, intraoperative retinal photocoagulation, vitreous hemorrhage, macular detachment, fibrovascular membrane, intraocular lens implantation, MIVS) except the stratification factor itself. All the results of stratified analysis are shown in **Table 4**. Due to the limitation of sample size, many variables cannot be counted after stratification.

## Discussion

This study demonstrated that compared with oral hypoglycemic medication, only insulin treatment had an association with the reduced risk of postoperative DME in all adjusted models. These stable and consistent results indicated that long-term insulin therapy seems to be beneficial in reducing the risk of postoperative DME. While the association between insulin therapy and reduced risk of postoperative DME seems to have nothing to do with HbA1c. In the stratified analysis, we can see that between different stratifications (age, gender, different HbA1c, gas tamponade, hypertension, posterior vitreous detachment, reoperation, MIVS, postoperative complications), oral medication plus insulin may exacerbate DME, although there is no statistical difference, the direction of the effect value is biased towards exacerbating DME. It may be necessary to increase the sample size to further verify whether the results are stable between different stratifications and whether the stratification will lead to different results.

Most previous studies (14–21) have confirmed that insulin might contribute to a higher risk of DME, which is different from the results of postoperative DME in this study. We think that there are the following reasons: Firstly, differences in the study population may lead to such inconsistent results. The population of previous studies included patients with DME from all stages of DR (14, 18, 19, 30). In this study, the population is all patients with PDR who have undergone PPV treatment, and their DR stages are comparable. We believe that it is more reasonable to study the association between different hypoglycemic regimens and DME under the unified classification of diabetes and the DR stage. Secondly, compared with DME without PPV surgery, postoperative DME has been relieved within a certain period by removing vitreoretinal traction and clearing various cytokines including VEGF which had a synergistic effect with insulin for higher risks of DR development in patients using insulin (32). Thirdly, insulin therapy is generally advocated when oral medications are insufficient in controlling glycemic. In the early stage of insulin treatment, there may be a series of edema reactions in the body due to drug switching or glycemic control (20, 35). While chronic insulin therapy, compared with oral hypoglycemic agents, does not modify the anatomic or functional effectiveness of DME treatment (23, 36, 37). In this study, the PDR patients treated with insulin had a longer course of diabetes. Long-term insulin therapy might reduce the risk of postoperative DME by lowering glycemia and HbA1c persistently which is termed metabolic memory (29). Metabolic memory was shown to persist through 10 years of follow-up and intensive glycemic control could reduce the risks of clinically significant DME in type 1 DM patients (29). In this study, due to metabolic memory, the advantage of strict glycemic control in the onset of postoperative DME might be reflected. At the same time, we found that although there is no statistical difference, HbA1c was lower in patients with oral medication than in patients with oral medication plus insulin and insulin. Apart from HbA1c, there may be other mechanisms to explain this phenomenon.

Except for insulin, different oral hypoglycemic medications have different effects on DME (27, 28). Most previous studies have shown that TZDs contributed to the increased risk of DME (26–28, 38). While another study (39) demonstrated that TZDs do not cause subclinical DME in a demographically diverse T2DM population, whether the TZDs are combined with other agents or not. Therefore, the relationship between TZDs and DME is still inconsistent. In addition to TZDs, sulphonylureas treatment was also related to a high risk of DME (14), while metformin did not have a relationship with the occurrence of clinically significant DME (30). Taken together, because different hypoglycemic medications may have different effects on DME and their mechanisms on DME are not fully understood, it is very important to clarify the proportion of different hypoglycemic mechanisms in hypoglycemic medications. Poor glycemic control applied by oral hypoglycemic medications might contribute to a higher risk of DME.

PPV itself has been widely used to treat tractional or refractory DME (40–43), which relieves DME through multiple mechanisms, including the elimination of traction factors (44), improving intravitreal oxygenation, removing pathological cytokines (such as VEGF) in the vitreous cavity, and accelerating the half-life of intravitreal cytokines (45). Whereas the low incidence of postoperative DME after PPV, there are few studies on the risk factors for postoperative DME in patients with PDR. A study (46) revealed that central macular thickness of the vitrectomized eyes was significantly correlated with atherogenic index of plasma, total cholesterol, low-density lipoprotein cholesterol, and uric acid. Kojima et al. (47) found that in DME patients, preoperative low HbA1c and postoperative pseudophakia were independently associated with the decrease in foveal thickness of the vitrectomized eyes. The two studies aforementioned confirmed that systemic factors might play an important role in the pathogenesis of postoperative DME. Based on clinical experience and observation, we believed that early postoperative DME may be related to surgical procedures (48, 49) and inflammatory reactions related to surgical procedures (49). In the late stage of postoperative DME may be related to systemic factors such as preoperative HbA1c (47), and the ocular accumulation of cytokines related to systemic factors may be another explanation. Therefore, we speculated that glucose control as one of the systemic factors may attribute to postoperative DME most. In the present secondary analysis, the association between the different regimens for glucose control and postoperative DME is circumstantial evidence for our hypotheses. We will further validate the hypotheses in our prospective PDR cohort (50).

Our study has some strengths. First, we treated different hypoglycemic regimens as a categorical variable and tested the P for trend, which is useful in evaluating the robustness of data analysis. Second, this was a historical cohort study and was thus susceptible to potential confounding. However, we used strict statistical adjustment to minimize the effect of residual confounders. Last, the results of present study provide a new idea of the relationship between DME secondary to vitrectomy with different hypoglycemic drugs, which is worthy of further exploration in future research.

However, the current study has several limitations. First, because of the retrospective cohort design, the proportion of different hypoglycemic mechanisms in oral DM medications and other drugs which might reduce the risks of DME such as aspirin and angiotensin-converting enzyme inhibitor is not clear. A prospective cohort study aiming at the different hypoglycemic regimens on postoperative DME is required. Second, because it is a retrospective cohort study, whether the presence of preoperative DME secondary to media opacification, and the definition and macular thickness of postoperative DME is unclear. The incidence of postoperative DME may be underestimated. It is noteworthy that the potential lack of information on outcomes resulting from such errors would bias toward the null and thus results in an underestimation of the association between different hypoglycemic regimens and postoperative DME. Third, the specific measurement time of postoperative DME is not known, and the pathogenesis of DME at different time points may be different. Besides, the period of oral medication or insulin that was prescribed for glycemic control should be considered. Finally, the generalization of the results may be limited, as the study population is all Japanese and the study had limited power to identify statistically significant differences in different stratifications, because of the small number of subjects included.

In conclusion, our results of the secondary analysis should be interpreted as a significant association between insulin treatment and reduced risks of DME in Japanese PDR patients with PPV surgery, compared with oral medications. Well glycemic control with longstanding insulin therapy may be beneficial to reduce the risks of postoperative DME in PDR patients with T2DM. Our investigation calls for large-scale and long-term prospective clinical studies for a full evaluation of the exact role of insulin in the progression of postoperative DME.

## Data Availability Statement

The datasets presented in this study can be found in online repositories. The names of the repository/repositories and accession number(s) can be found in the article/**Supplementary Material**.

## Ethics Statement

This study was performed by Japanese researcher Nishi et al. at the Yamagata University Hospital, Yamagata, Japan. In the previously published article [34], Nishi et al. have clearly stated that: the study was performed following the Declaration of Helsinki and reviewed and approved by the Ethics Committee of the Yamagata University Faculty of Medicine (approval number: H26-21). All data were fully anonymized before we accessed them and the IRB waived the requirement for informed consent. Written informed consent for participation was not required for this study in accordance with the national legislation and the institutional requirements.

## Author Contributions

Conceptualization:CL, YZ, MZ; Formal analysis: CL; Validation: YZ; Supervision: MZ; Writing – original draft: CL, YZ, MZ; Writing – review & editing: CL, YZ, MZ. All authors contributed to the article and approved the submitted version.

## Funding

This work was supported by 1.3.5 project for disciplines of excellence, West China Hospital, Sichuan University (ZYJC21025), and the Sichuan Provincial Science and Technology Support Project (no.2019YJ0129), and China Postdoctoral Science Foundation (2021M692273).

## Acknowledgments

Thanks for the comprehensive raw data provided by professor Koichi Nishitsuka’s team. They completed the entire study. They are (the rankings and institutions of these researchers were ranked according to the “reference (34)“) Katsuhiro Nishi, Koichi Nishitsuka (corresponding author) (Department of Ophthalmology and Visual Sciences, Yamagata University Faculty of Medicine, Yamagata, Yamagata, Japan), Teiko Yamamoto, Hidetoshi Yamashita.

## Conflict of Interest

The authors declare that the research was conducted in the absence of any commercial or financial relationships that could be construed as a potential conflict of interest.

## Publisher’s Note

All claims expressed in this article are solely those of the authors and do not necessarily represent those of their affiliated organizations, or those of the publisher, the editors and the reviewers. Any product that may be evaluated in this article, or claim that may be made by its manufacturer, is not guaranteed or endorsed by the publisher.
